# Newly identified pathogens in periodontitis: evidence from an association and an elimination study

**DOI:** 10.1080/20002297.2023.2213111

**Published:** 2023-05-27

**Authors:** Eduardo Lobão Veras, Nídia Castro dos Santos, João Gabriel S. Souza, Luciene C. Figueiredo, Belen Retamal-Valdes, Valentim A. R. Barão, Jamil Shibli, Martinna Bertolini, Marcelo Faveri, Flavia Teles, Poliana Duarte, Magda Feres

**Affiliations:** a Department of Periodontology, Dental Research Division, Guarulhos University, Guarulhos, SP, Brazil; b The Forsyth Institute, Cambridge, MA, USA; c Department of Dental Research, Dental Science School (Faculdade de Ciências Odontológicas - FCO), Montes Claros, Brazil; d Department of Prosthodontics and Periodontology, Piracicaba Dental School, University of Campinas (UNICAMP), Piracicaba, Brazil; e Department of Periodontics and Preventive Dentistry, School of Dental Medicine, University of Pittsburgh, Pittsburgh, PA, USA; f Center for Innovation & Precision Dentistry, School of Dental Medicine, School of Engineering and Applied Sciences, University of Pennsylvania, Philadelphia, PA, USA; g Department of Oral Medicine, Infection and Immunity, Harvard School of Dental Medicine, Boston, MA, USA

**Keywords:** Periodontitis, etiology, new pathogens, periodontal treatment, oral microbiology, Microbiota

## Abstract

We assessed the level of evidence for the presence of new periodontal pathogens by (i) comparing the occurrence of non-classical periodontal taxa between healthy vs. periodontitis patients (Association study); (ii) assessing the modifications in the prevalence and levels of these species after treatments (Elimination study). In the Association study, we compared the prevalence and levels of 39 novel bacterial species between periodontally healthy and periodontitis patients. In the Elimination study, we analyzed samples from periodontitis patients assigned to receive scaling and root planing alone or with metronidazole+ amoxicillin TID/ 14 days. Levels of 79 bacterial species (39 novel and 40 classic) were assessed at baseline, 3 and 12 months post-therapy. All samples were analyzed using Checkerboard DNA-DNA hybridization. Out of the 39 novel species evaluated, eight were categorized as having strong and four as having moderate association with periodontitis. Our findings suggest strong evidence supporting *Lancefieldella rimae*, *Cronobacter sakazakii*, *Pluralibacter gergoviae*, *Enterococcus faecalis*, *Eubacterium limosum*, *Filifactor alocis*, *Haemophilus influenzae*, and *Staphylococcus warneri*, and moderate evidence supporting *Escherichia coli*, *Fusobacterium necrophorum*, *Spiroplasma ixodetis*, and *Staphylococcus aureus* as periodontal pathogens. These findings contribute to a better understanding of the etiology of periodontitis and may guide future diagnostic and interventional studies.

**Figure:**
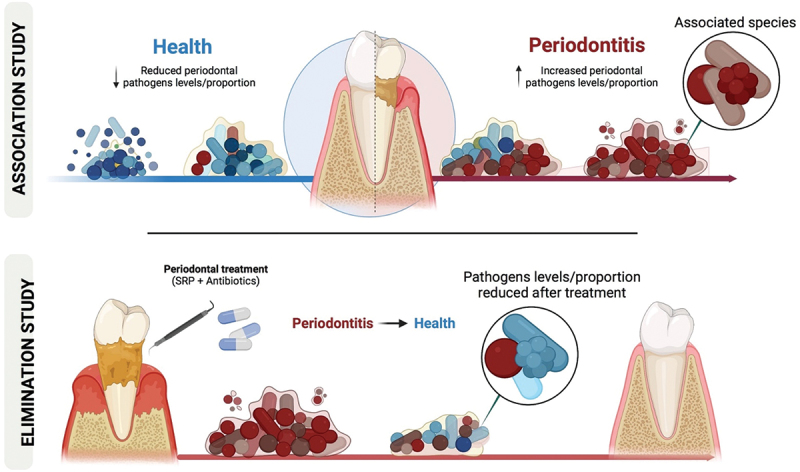

## Introduction

The occurrence and progression of periodontitis involve a complex interaction between the periodontal microbiome and the immune system [1,2]. As a disease associated with polymicrobial dysbiosis, effective prevention, and management of periodontitis is dependent on the identification of microorganisms associated with its onset and progression.

Socransky et al. [3] used the Checkerboard DNA-DNA hybridization technique to evaluate the composition of subgingival biofilm samples (*n* = 13,261) from 160 volunteers with periodontitis and 25 with periodontal health. The authors described three microbial complexes (green, purple, and yellow) associated with periodontal health and two groups of microorganisms (red and orange) strongly associated with clinical signs of disease. The red complex was composed of *Porphyromonas gingivalis, Tannerella forsythia*, and *Treponema denticola* while the orange complex congregated ten species from different genera, such as *Fusobacterium, Prevotella*, and *Campylobacter*. Importantly, a subsequent study demonstrated that four *Actinomyces* species were also closely related to periodontal health [4]. These studies have greatly contributed to increasing knowledge of the periodontal microbial ecology. Furthermore, the 40 bacterial species from these microbial complexes have been successfully used as biological markers in studies testing the effects of periodontal therapies on reestablishing a health-associated microbiome [5,6,7–11,12,13–15].

As growing knowledge on the oral microbiome composition emerges, especially through next-generation sequencing (NGS), other species have been suggested as possible bacterial pathogens [15,16,17–19,20,21]. A systematic review pointed out that bacteria from the phyla *Bacteroidetes, Firmicutes, Proteobacteria, Saccharibacteria, Spirochaetes*, and *Synergistetes* might be related to periodontal disease onset and progression [21].

The main criteria used to associate a certain microorganism with the etiology of infection are the postulates established by [22]. In the context of periodontal infections, the postulates modified by Socransky [23], have been largely used to establish a true periodontal pathogen, as follows: 1) Association with disease implies increased proportions or levels of the microorganism at sites of disease and reduced proportions or levels (or absence) in healthy sites or sites with other forms of disease; 2) Elimination of the organism is a critical test of its effect during disease by its elimination or reduction leading to disease suppression; 3) A pathogen should lead to an increased or decreased host immune response; 4) Animal models should replicate what is observed in human disease; 5) A putative microorganism should have particular pathogenic mechanisms.

Socransky [23] suggested that the most important studies to associate a microorganism with a given pathology are the Association and Elimination studies (postulates 1 and 2). Several Association studies have evaluated the possible involvement of newly identified species in the etiology of periodontitis, either by targeted or open-ended microbiological techniques, as shown by previous studies [15,21]. However, most studies evaluated few biofilm samples. In addition, Elimination studies aiming to determine changes in the levels or proportions of these species after periodontal treatment are scarce [15]. Combining Association and Elimination studies may help to identify new species involved in the etiology of periodontitis and understand the ecology of the oral cavity. Hence, we determined the level of evidence for novel periodontal pathogens by (i) comparing the occurrence of 39 novel bacterial species in healthy and periodontitis patients (Association study) and (ii) assessing their behavior after periodontal treatment (Elimination study).

## Materials and methods

This investigation included two different sections: (i) an Association and (ii) an Elimination study. The Association study aimed at comparing the prevalence and levels of 39 novel bacterial species between periodontally healthy and diseased patients. The Elimination study was designed as a randomized controlled trial to test changes in the prevalence and levels of these species after treatment.

### Study population and inclusion and exclusion criteria: Association and Elimination Studies

Patients with stages III and IV, grades B and C, generalized periodontitis, and periodontally healthy individuals were selected from the Periodontal Clinic at Guarulhos University (Guarulhos, SP, Brazil). Informed consent was provided by each patient after a thorough explanation of the risks and benefits of their participation in the study. The study was approved by the human subjects ethics board of Guarulhos University (Protocol 437.155).

Inclusion criteria for patients with periodontitis were: at least 6 teeth with≥1 interproximal non-contiguous sites with probing depth (PD) and clinical attachment level (CAL) ≥5 mm; at least 15 teeth; ≥30% of sites with bleeding on probing (BoP) and PD and CAL≥4 mm; ≥30 years old.

Inclusion criteria for periodontally healthy patients were: ≥15 teeth; no site with PD and CAL>3 mm; <10% of sites with BoP; ≥30 years old.

Exclusion criteria for all patients were: previous periodontal treatment; smokers or former smokers (≤5 years); pregnancy or lactation; any systemic disease that can affect the pathogenesis of periodontal diseases need for antibiotic prophylaxis; current orthodontic treatment; use of antibiotics in the previous 6 months; allergy to metronidazole (MTZ) or penicillin for patients with periodontitis.

### Clinical assessment: association and Elimination Studies

Two examiners participated in a calibration exercise to assess intra- and inter-examiner variability for PD and CAL measurements. Calibration was carried out according to Araujo et al. [24] and the standard error (SE) of measurement was calculated. The average level of intra- and inter-examiner agreement for the categorical variables was>93% (Kappa test). Clinical measurements were performed at baseline for periodontally healthy individuals and periodontitis patients (Association study), and at 3 and 12 months for patients with periodontitis (Elimination study). Mean CAL, PD, and the percentage of sites with plaque, gingival bleeding, BoP and suppuration (SUP) were assessed at 6 sites/teeth using a North Carolina periodontal probe (PCPUNC-BR 15 Hu-Friedy, Rio de Janeiro, RJ, Brazil).

### Microbial assessment: association and Elimination Studies

For patients with periodontitis, 9 subgingival biofilm samples per patient were collected at baseline, and at 3 and 12 months post-treatment, 3 from each of the following categories of sites: shallow (PD ≤3 mm), moderate (PD = 4–6 mm), and deep (PD ≥7 mm). For healthy patients, 9 randomly biofilm samples were collected from sites with PD ≤3 mm. After supragingival biofilm and calculus removal, subgingival plaque samples were collected with periodontal curettes (Gracey mini-five 11–12) and kept in pre-coded sterile microtubes with 150 μL TE buffer (10 mM Tris-HCL) (Invitrogen Life Technologies, Carlsbad, CA, USA). Then, 100 μL of 0.5 M NaOH solution was added. The microtubes were kept frozen (−20ºC) until the biofilm samples were analyzed using Checkerboard DNA-DNA hybridization [9,25,26].

Genomic probes were prepared for 39 ”novel” pathogens not yet been proven to be related to the etiology or progression of periodontitis. The selection of these potential pathogens was performed as follows:

1. Species that have been suggested as possible periodontal pathogens by Pérez-Chaparro et al. [21]. The authors reported that 34 newly identified bacterial species could be considered periodontal pathogens. As 22 of these are cultivable species, they could be analyzed by Checkerboard DNA-DNA hybridization. We obtained DNA from 9 of these 22 cultivable species and designed their probes. However, 3 of these bacterial species (*Eubacterium saphenum, Selenomonas sputigena*, and *Acinetobacter baumannii*) were eventually excluded because of nonfunctioning DNA probes. Thus, 6 of these bacterial species were evaluated in the present study: *Dialister pneumosintes, Enterococcus faecalis, Escherichia coli, Eubacterium brachy, Filifactor alocis*, and *Porphyromonas endodontalis.*
2. Bacterial species of medical importance and/or suggested by Dr. Sigmund Socransky and Dr. Flavia Teles (advisory expert opinion): seven species (*Lancefieldella rimae, Haemophilus influenzae, Klebsiella pneumoniae, Prevotella oris, Rothia dentocariosa, Staphylococcus aureus*, and *Staphylococcus epidermidis)* that had already been associated with the etiology of periodontitis in at least one previous publication were selected [15]. The other 26 microorganisms were selected for being pointed out as bacterial species of medical importance or related to extra-oral infections. *Streptococcus pneumoniae* was only included in the Elimination study for being identified in healthy volunteers only [27]. Therefore, 39 species were evaluated in the Association study and 40 species were assessed in the Elimination study.

### Checkerboard DNA-DNA hybridization

Microbial analyses were performed using Checkerboard DNA-DNA Hybridization according to Socransky and Haffajee [25], and Soares et al. [26] (Suppl. materials 1).

### Periodontal treatment: elimination study

After biofilm samples were collected, patients with periodontitis were randomly assigned to receive scaling and root planing (SRP) with or without systemic antibiotics. The control group received SRP+ placebos TID for 14 days, and the test group (MTZ+AMX) received SRP+ MTZ (400 mg) + amoxicillin (AMX, 500 mg), TID for 14 days. This was a double-blind, placebo-controlled interventional study. Microbiological monitoring of the 39 new periodontal pathogens and 40 classic periodontal species was conducted at baseline and at 3 and 12 months after therapy. SRP was performed in 4 to 6 appointments within 14 days by a trained periodontist using an ultrasonic device and curette. The antibiotic or placebo regimens began on the first SRP session. Patients were engaged in supportive periodontal therapy every 3 months, including supragingival plaque and calculus removal, subgingival scaling of sites with PD ≥4 mm, and oral hygiene instructions.

### Statistical analysis

#### Association study

Mean CAL and PD and mean percentage of sites with plaque, gingival bleeding, BoP, and suppuration were recorded for each patient and within each group. The bacterial levels and prevalence of the ‘new’ bacterial species were recorded from biofilm samples of patients with periodontal health and disease The mean levels (x10^5^) of each species were recorded per site, per patient, and per group. The same procedures were applied for microbiological data recorded from shallow sites (i.e. PD ≤3 mm) of both groups. The percentage of sites and patients colonized by each species was recorded. The differences between periodontally healthy and periodontitis groups were assessed by the Mann-Whitney test. The chi-square test was used to compare gender and the frequency of patients colonized by each bacterial species. Statistical significance was established as 5%. Microbiological analyses were performed with and without adjusting for multiple comparisons. A p-value of<0.00125 was applied for the adjusted comparisons, as proposed by Socransky et al. [28].

#### Elimination study

The ideal sample size to assure adequate power for this study was based on a difference of at least 4 sites with PD ≥5 mm [29] between groups and a standard deviation of 5 sites with PD ≥5 mm [9]. Considering a significance level of 5%, 20 subjects per group would be necessary to provide a power of 80%. Mean CAL and PD, the percentage of sites with gingival bleeding, plaque, BoP, and suppuration, and mean number of sites with PD ≥5 mm were recorded per patient and within the groups. Intragroup differences (from baseline to 1-year post-treatment) were assessed using the Wilcoxon test. Thedifferences between treatment groups at each time point were assessed by the Mann-Whitney test. The percentage of patients achieving the clinical endpoint for treatment (≤4 sites PD ≥5 mm [29]; 1-year post-therapy was evaluated using the Chi-square test.

Microbiological data from subgingival biofilm samples of both therapeutic groups were expressed as bacterial loads (levels/count), prevalence, and proportion. The mean levels (x10^5^) and prevalence of each new species were recorded per site, per patient, and per group at each time point. Intragroup differences (baseline, 3 months, and 1 year) for these microbiological parameters were assessed by the Friedman test. Differences between treatment groups at each time point were assessed by the Mann-Whitney test. The mean proportions of the microbial complexes [3] between baseline and 1 year within each treatment group were compared using the Wilcoxon test, while the mean proportions of the microbial complexes between groups at each time point were compared by the Mann-Whitney test. Statistical significance was established as 5%. Microbiological analyses were performed with and without adjustment for multiple comparisons. A p-value of<0.00125 was considered for multiple comparisons, as proposed by Socransky et al. [28].

### Classification of potential “new” periodontal pathogens

Results from the Association and the Elimination studies were grouped, and bacterial species were categorized into 2 levels of evidence for the status of ‘periodontal pathogen’ according to the following criteria:

1. Strong evidence: when the species was significantly elevated in periodontitis patients when compared to periodontally healthy individuals considering the following 5 parameters: % of patients colonized (i); % of colonized sites considering all sites (ii) or only shallow sites (iii); mean bacterial counts considering all sites (iv) or only shallow sites (v). Besides that, the species should be reduced after treatment according to the following parameters: percentage of colonized sites (i) and mean levels (ii), by at least one of the treatment protocols. For the analysis of ‘shallow sites’, adjusted and non-adjusted statistical significance for multiple comparisons were considered, whereas for the other parameters only adjusted significances were considered.
2. Moderate evidence: when the species was elevated in periodontitis patients when compared to healthy individuals for at least 4 of the 5 abovementioned parameters, considering adjusted and non-adjusted statistical significances. Besides that, the species should be reduced for the following parameters: percentage of colonized sites (i) and mean levels (ii), by at least one of the treatment protocols.

## Results

### Association Study

Demographic and clinical baseline characteristics of healthy and periodontitis groups are presented in Table 1. All clinical parameters were significantly higher forpatients with periodontitis (*n* = 40) than for periodontally healthy (*n* = 17) individuals (*p* < 0.05).

**Table 1.** Association study: Clinical and demographic parameters of patients at baseline.

	Groups		
	Healthy (*n* = 17)	Periodontitis (*n* = 40)	
Variables	Mean ± SD	Mean ± SD	*p-value*
Gender (F/M)	10/7	18/12	>0.05#
Age (years)	40.6 ± 4.1	45.6 ± 8.1	0.5674*
PD (mm)	1.96 ± 0.6	3.82 ± 0.71	0.0007*
CAL (mm)	1.04 ± 0.5	4.23 ± 0.93	0.0099*
sites with:			
Visible plaque (%)	34.5 ± 9.9	84.1 ± 12.3	0.0103*
Gingival bleeding (%)	4.5 ± 2.9	29.2 ± 11.1	0.0029*
BOP (%)	6.1 ± 3.2	81.1 ± 14.1	0.0004*
SUP (%)	0.0 ± 0.0	2.5 ± 3.2	0.0000*

SD, Standard deviation; PD, Probing depth; CAL, Clinical attachment level; BOP, Bleeding on probing; SUP, Suppuration. *Mann-Whitney test. # Chi-square test.

**Figure 1.**
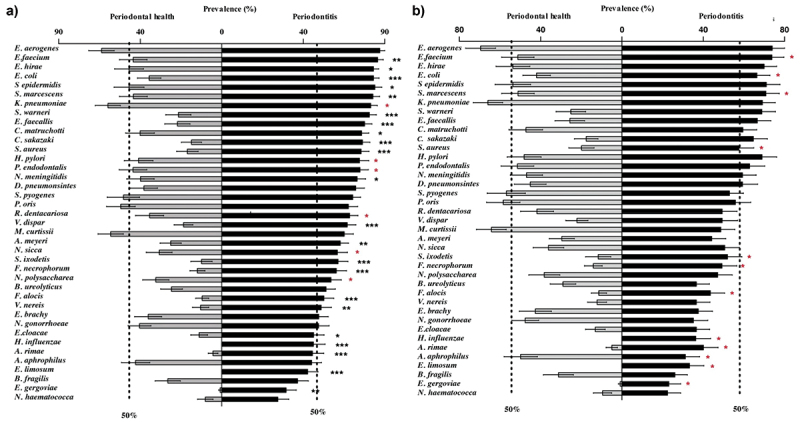
Mean percentage of all evaluated sites (a) and of shallow sites (b) colonized by the 39 ‘new’ bacterial species in periodontal health and disease. The species are presented in descending order according to their prevalence in the periodontitis group. (*p < 0.05, Mann-Whitney test). (* in black) indicates statistical significance adjusted for multiple comparisons; (* in red) indicates unadjusted statistical significance.

Table 2 depicts the 17 bacterial species that colonized a higher percentage of subjects with periodontitis than with periodontal health (*p* < 0.05). The mean percentage of all sites colonized by the 39 bacterial species evaluated is presented in Figure 1. When all sites were analyzed (Figure 1a), 22 species were observed in a significantly higher percentage of sites in patients with periodontitis than in periodontally healthy individuals (*p* < 0.05). When only shallow sites were considered (Figure 1b), *E. faecalis, Staphylococcus warneri*, and *Cronobacter sakazakii* colonized a higher percentage of sites of patients with periodontitis (adjusting for multiple comparisons). Eleven species (*L. rimae, Pluralibacter gergoviae, Enterococcus faecium, E. coli, Eubacterium limosum, F. alocis, Fusobacterium necrophorum, H. influenzae, Serratia marcescens, Spiroplasma ixodetis*, and *S. aureus)* were detected in a higher percentage of sites and *Aggregatibacter aphrophilus* in a lower percentage of sites of periodontitis patients than in healthy individuals, when the non-adjusted analysis was pondered.

**Table 2.** Species colonizing a statistically significantly higher percentage of patients with periodontitis than periodontally healthy individuals.

	Groups		
Microrganismos	Healthy (*n* = 17)	Periodontitis (*n* = 40)	p-value
*Actinomyces meyeri*	76.5	97.5	0.0242**
*Lancefieldella rimae*	23.5	80.0	0.0002*
*Cronobacter sakazakii*	58.8	100	<0.0001*
*Enterobacter cloacae*	41.2	80.0	0.0060**
*Enterobacter gergoviae*	5.9	67.5	<0.0001*
*Enterococcus faecallis*	58.8	97.5	<0.0001*
*Escherichia coli*	82.4	100	0.0200**
*Eubacterium limosum*	0.0	82.5	<0.0001*
*Filifactor alocis*	47.1	90.0	0.0010*
*Fusobacterium necrophorum*	52.9	90.0	0.0035**
*Haemophilus influenzae*	0.0	75.0	<0.0001*
*Spiroplasma ixodetis*	41.2	90.0	0.0020**
*Staphylococcus aureus*	58.8	95.0	0.0018**
*Staphylococcus warneri*	70.6	100	<0.0001*
*Streptococcus pyogenes*	82.4	100	0.0232**
*Veillonella díspar*	64.7	97.5	0.0019**
*Vibrio nereis*	47.1	87.5	0.0023**

*p* < 0.05 means statistically significant differences between groups by the Chi-square test, with (*) and without (**) adjustments for multiple comparisons.

**Figure 2.**
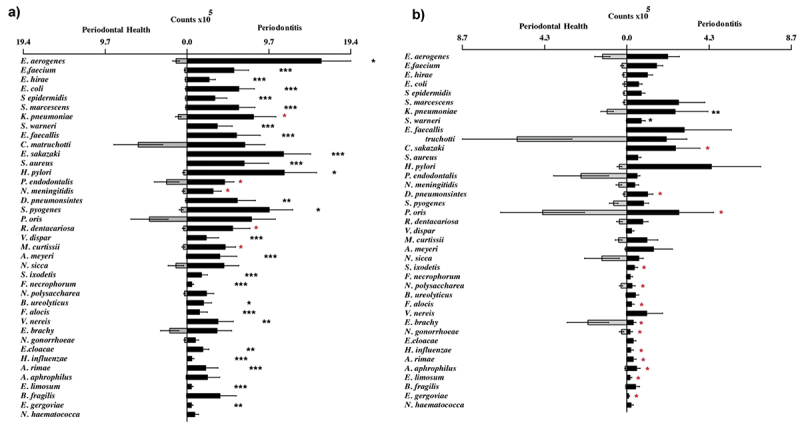
Mean counts of the 39 ‘new’ bacterial species in periodontal health and disease of all evaluated sites (a) and in shallow sites (b). The species are listed in descending order according to the average percentage of colonized sites in the periodontitis group (Figure 1). The statistical significance of differences between groups was assessed using the Mann-Whitney test (*p* < 0.05). (* in black) indicates statistical significance adjusted for multiple comparisons; (* in red) indicates unadjusted statistical significance.

The levels of 30 species (25 after adjustments for multiple comparisons) were higher in periodontitis than in healthy patients considering all sites (*p* < 0.05). The levels of 15 species (2 after adjustments for multiple comparisons) were higher in periodontitis compared to healthy patients considering the shallow sites only (Figure 2).

### Elimination Study

Table 3 presents the demographic characteristics and baseline clinical parameters of the groups, before and after periodontal therapy. Clinical parameters did not differ between treatment groups at baseline (*p* > 0.05). At 1-year post-therapy, improved clinical parameters were observed for both groups. However, the group that received SRP + MTZ + AMX showed more striking clinical benefits than the placebo group (*p* < 0.05). The mean number of sites with PD ≥5 mm 1 year after periodontal therapy was 14.5 ± 10.2 for the placebo group and 4.1 ± 4.8 for the SRP+MTZ+AMX group (*p* < 0.05, adjusted for baseline values). 70% of patients in the test group achieved the clinical endpoint for periodontal treatment (≤4 sites with PD ≥5 mm [29]; as opposed to 10% of those in the placebo group.

**Table 3.** Elimination study: Demographic characteristics and clinical parameters of both treatment groups, before and after periodontal therapy.

		Treatment groups		
Variables	Time	SRP (*n* = 20)	SRP+ MTZ + AMX (*n* = 20)	p-value
Gender (F/M)	Baseline	16/4	12/8	0.300#
Age (Mean ± SD, years)	Baseline	44.8 ± 8.9	46.2 ± 8.9	0.473*
PD (Mean ± SD, mm)	Baseline	3.88 ± 0.64	3.70 ± 0.66	0.514*
CAL (Mean ± SD, mm)	Baseline	4.25 ± 0.74	4.15 ± 0.89	0.375*
Mean number (± SD) of sites with PD ≥5 mm	Baseline	38.8 ± 21.6 ^A^	33.0 ± 20.0 ^A^	0.089*
	1 year	14.5 ± 10.2 ^B, a^	4.1 ± 4.8 ^B, b^	<0.0001*
Number and % of patients achieving the clinical endpoint for treatment, i.e. < 4 sites PD > 5 mm [29].	1 year	2 (10.0%)^a^	14 (70.0%)^b^	<0.0001#

The statistical significance of differences between groups at baseline and at 1-year post-treatment was assessed by the Mann-Whitney (*) test or the Chi-square test (#). Different lowercase letters indicate significant differences between groups, *p* < 0.05.

The statistical significance of differences within each group for the mean number of sites with PD ≥5 mm (between baseline and 1 year) was assessed by the Wilcoxon test. Different capital letters indicate significant differences between time points, *p* < 0.05.

SRP, scaling and root planing; MTZ, metronidazole; AMX, amoxicillin; PD, probing depth, CAL, clinical attachment level; SD: Standard deviation.

**Figure 3.**
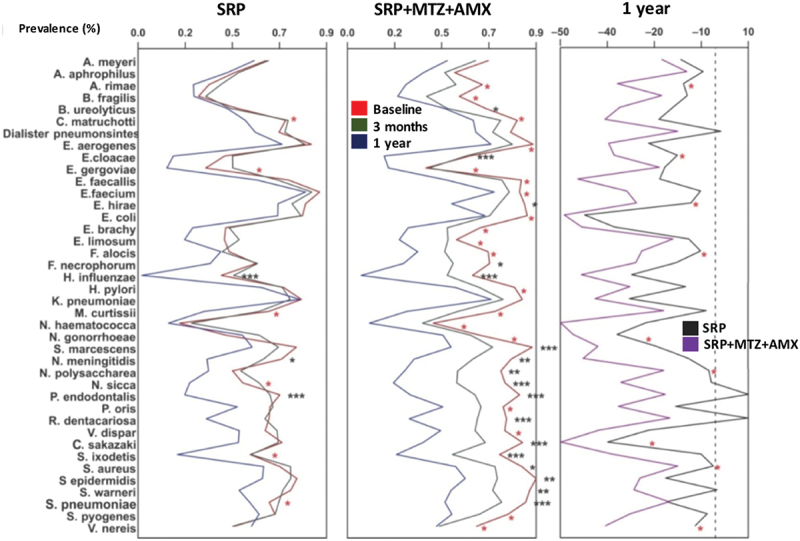
Average of periodontal sites colonized (prevalence) by the new pathogens evaluated for the treatment group SRP or SRP+MTZ+AMX at baseline, after 3 months and 1 year. Comparison between treatment groups after 1 year. SRP – Scaling root planing; MTZ – Metronidazole; AMX – Amoxicillin. The statistical significance of differences between groups was assessed using the Mann-Whitney or Friedman test (*p* < 0.05). (* in black) indicates statistical significance adjusted for multiple comparisons; (* in red) indicates unadjusted statistical significance.

Figure 3 displays the effects of therapies (Elimination study) on the mean number of sites colonized by the ‘new’ species at 3 months and 1 year, and the comparison between groups between baseline and 1 year. For the analysis adjusted for multiple comparisons, SRP significantly reduced 3 species (*H. influenzae*, *N. meningitidis*, and *P. endodontalis*), and SRP+MTZ+AMX, 17 species (*Campylobacter ureolyticus, C. sakazakii, E. cloacae, Enteroccocus hirae, F. necrophorum, H. influenzae, N. meningitidis, N. polysaccharea, Neisseria sicca, P. endodontalis, R. dentocariosa, S. marcescens, S. ixodetis, S. aureus, S. epidermidis, S. warneri*, and *S. pneumoniae)*. When the groups were compared at 1 year, patients in the SRP+MTZ+AMX presented fewer sites colonized by 9 species (*L. rimae, C. sakazakii, E. hirae, F. alocis, N. gonorrhoeae, N. polysaccharea, S. aureus, V. nereis*, and *E. cloacae*) than those in the SRP group. However, these differences were only observed for the non-adjusted comparisons.

**Figure 4.**
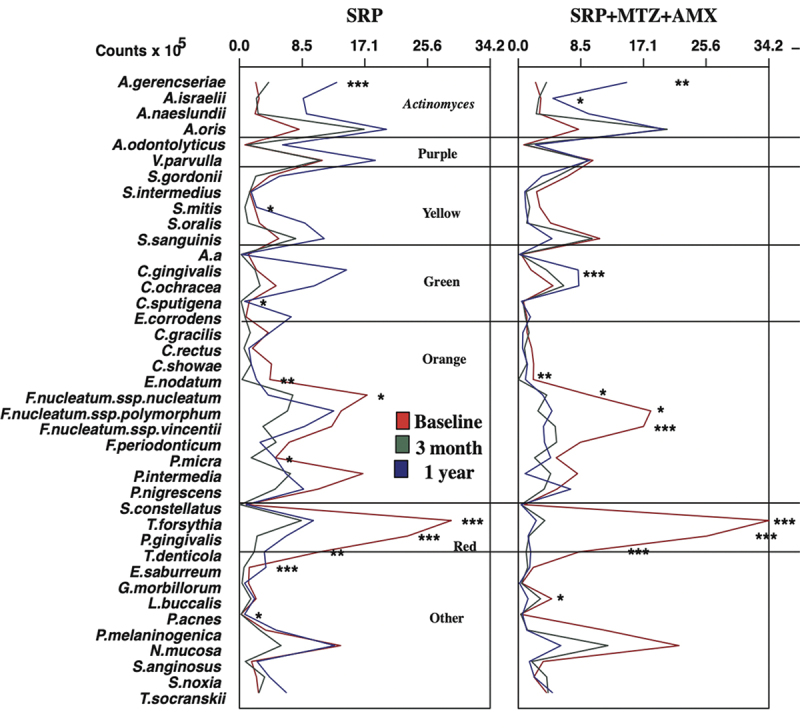
Mean levels of the classic 40 bacterial species 3Mean levels of the 40 classic bacterial species from the microbial complexes [3] at baseline, 3 months and 1 year. SRP – Scaling root planning; MTZ – Metronidazole; AMX – Amoxicillin. The statistical significance of differences within eacg treatment group over time was assessed using the Friedmantest (*p* < 0.05) (* in black) indicates statistical significance adjusted for multiple comparisons; (*in red) indicates unadjusted statistical significance.

**Figure 5.**
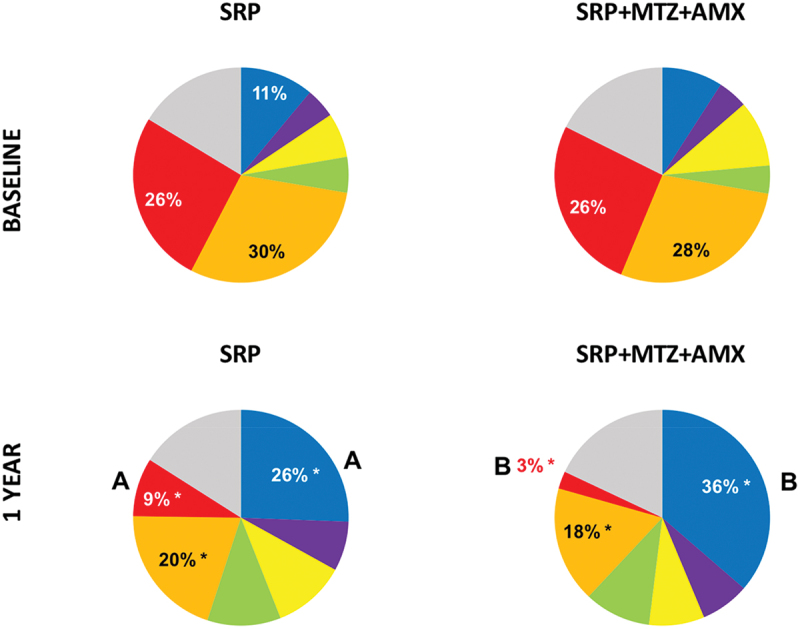
Mean proportions of the microbial species described by Socransky et al. [3] in the two treatment groups at baseline and 1 year. The significance of differences between groups was determined by the Mann-Whitney test (different letters = p < 0.05), and within each group (between the two time points) by the Wilcoxon test (*p < .05).

The effects of treatments on the individual mean levels of the 40 classic bacterial species from the microbial complexes [3] are presented in Figure 4. Both treatments reduced the individual levels of pathogens from the red and orange complexes and led to an increase in several host-compatible species. Nonetheless, the reduction in counts of the three red complex pathogens was more striking in the antibiotic group. The mean proportions of the microbial complexes [3]at baseline and 1 year are presented in Figure 5. This analysis includes the 40 bacterial species from the traditional Checkerboard DNA-DNA hybridization panel [26]. The proportions of orange and red complexes were significantly reduced by both treatments and the *Actinomyces* species increased (*p* < 0.05). At 1 year, patients treated with adjunctive MTZ + AMX had lower proportions of red complex (3%) and higher proportions of *Actinomyces* (36%), in comparison with those treated with SRP-only (9% and 26%, respectively). As a comparison, the mean proportions of all bacterial species evaluated in this study are presented in Supplementary Figure 1. Some classical and putative periodontal pathogens were among the species present in the highest proportions, such as *T. forsythia, P. gingivalis*, and several *Fusobacterium* species.

## Classification of potential “new” periodontal pathogens

Table 4 Species categorized in the two levels of evidence – Strong or Moderate – for the status of ‘periodontal pathogen’. Eight species were included in the Strong evidence category (*L. rimae*, *C. sakazakii*, *E gergoviae*, *E. faecalis, E. limosum*, *F. alocis, H. influenzae*, and *S. warneri*) while four species were categorized as Moderate evidence (*E. coli*, *F. necrophorum*, *S. ixodetis*, and *S. aureus*). Table 5 presents the summary of key findings from Association and Elimination studies used to determine the levels of evidence.

**Table 4.** Classification of potential ‘New’ periodontal pathogens according to the level of association with periodontitis.

	Species	ATCC Strain
Strong	*Lancefieldella rimae*	49626
	*Cronobacter sakazakii*	12868
	*Enterobacter gergoviae*	33028
	*Enterococcus faecallis*	29212
	*Eubacterium limosum*	8486
	*Filifactor alocis*	35896
	*Haemophilus influenzae*	33533
	*Staphylococcus warneri*	27836
Moderate	*Escherichia coli*	10799
	*Fusobacterium necrophorum*	25286
	*Spiroplasma ixodetis*	33835
	*Staphylococcus aureus*	33591

Strong evidence: when the species was significantly elevated in periodontitis patients when compared to periodontally healthy individuals considering the following 5 parameters: % of patients colonized (i); % of colonized sites considering all sites (ii) or only shallow sites (iii); mean bacterial counts inconsidering all sites (iv) or only shallow sites (v). Besides that, the species should be reduced aftertreatment according to the following parameters: percentage of colonized sites (i) and mean levels (ii), by at least one of the treatment protocols. For the analysis of ‘shallow sites’, adjusted and non-adjusted statistical significance for multiple comparisons were considered, whereas for the other parameters only adjusted significances were considered.

*For the analysis of ‘shallow sites’, adjusted and non-adjusted statistical significance for multiple comparisons were considered, whereas for the other parameters only adjusted significances were considered.

Moderate evidence: when the species was elevated in periodontitis patients when compared to healthy individuals for at least 4 of the 5 abovementioned parameters, considering adjusted and non-adjusted statistical significances. Besides that, the species should be reduced for the following parameters: percentage of colonized sites (i) and mean levels (ii), by at least one of the treatment protocols.

**Table 5.** Summary of key findings from Association and Elimination studies.

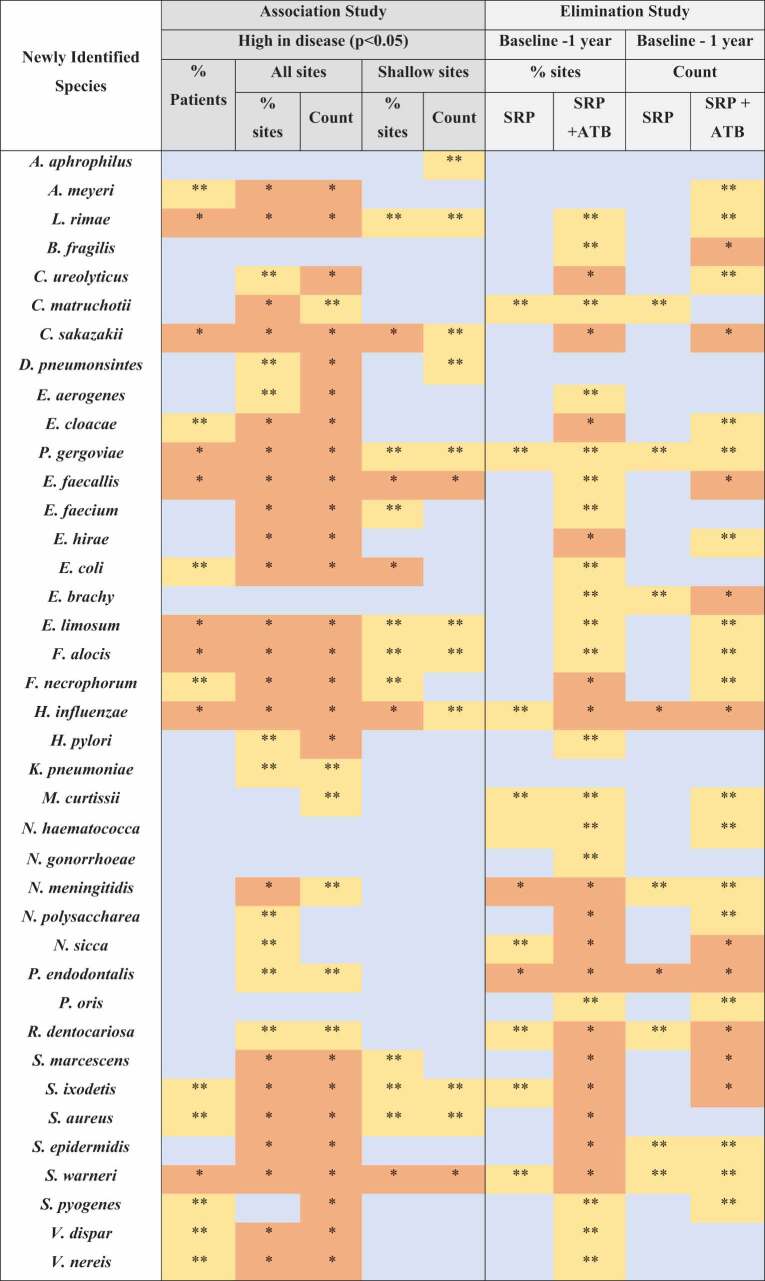

Adjusted statistical significance for multiple comparisons 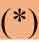; Unadjusted statistical significance 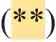; No difference 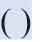 % sites: percentage of sites colonized; % patients: percentage of patients colonized; Baseline −1 year: bacterial species significantly reduced between baseline and 1-year post-therapies; SRP: scaling.

## Discussion

The data of this study suggested the association of 12 new bacterial species with the etiopathogenesis of periodontitis, with strong or moderate evidence. The bacterial species that showed strong evidence to be potential novel pathogens were *L. rimae, C. sakazakii, P. gergoviae, E. faecalis, E. limosum, F. alocis, H. influenzae*, and *S. warneri*; and those with moderate evidence were *E. coli, F. necrophorum, S. ixodetis*, and *S. aureus*.

Ecological studies have revealed that levels and proportions of a microorganism in periodontal pockets are more relevant to understand their role in health and disease than their mere presence/absence [4,30,31]. Thus, it is noteworthy that among the eight species showing strong association with periodontitis, three - *C. sakazakii, E. faecalis, and S. warneri* - were detected at higher levels and colonized a higher percentage of sites in patients with periodontitis than in healthy ones, when ‘all sites’ and only ‘shallow sites’ were evaluated. In addition, they were found at very low levels and prevalence in healthy volunteers [32].

*Sakazakii* is a gram-negative rod that belongs to the *Enterobacteriaceae* family and is not part of the normal microbiota of the human/animal oral cavity or gastrointestinal tract. This microorganism is recognized as an opportunistic pathogen of food origin and has been related to cases of neonatal meningitis [33,34]. In periodontics, C. sakazakii has been found in the subgingival biofilm of HIV-positive patients with necrotizing periodontitis [35]. However, this species had not yet been associated with periodontitis in systemically healthy patients [15,21]. Therefore, this is new information that deserves further assessment.

*Warneri* is a coagulase-negative Staphylococcus (CNS) commonly present in human mucous membranes and epithelial microbiota. This microorganism has been considered a potential new pathogen in non-oral severe infections [36,37]; but has rarely been studied in periodontology. The epidemiology and the pathogenesis of *S. warneri* are still unclear [38,39]. Colombo et al. [40] detected this species in subgingival biofilm samples in patients refractory to conventional periodontal treatment. These authors identified other bacterial species not commonly found in the oral cavity, including *A. baumannii*, *Gemella haemolysans*, *Pseudomonas aeruginosa*, and *E. faecalis* – a species also strongly associated with periodontitis in the current study.

*Faecalis* is a common commensal microorganism of the human gastrointestinal tract but may be an important opportunistic pathogen in other parts of the body. It is the third most frequent pathogen isolated from bacteremia, causing most of the postoperative infections in intensive care units [41,42]. Regarding oral infections, this species has been associated with cases of root canal treatment failure [43–48]. Several Association studies showed a relationship between *E. faecalis* and periodontitis in Brazilian patients [40,49,50], which could be an example of geographical specificity. Chidambar et al. [51] also reported a higher prevalence of *E. faecalis* in subgingival plaque samples of Indian patients with periodontitis. Those studies corroborate historical data showing subgingival *E. faecalis* occurring in 1% of early-onset periodontitis patients and in approximately 5% of adults with periodontitis [52]. While *E. faecalis* were suggested to populate periodontal pockets as superinfecting organisms, this theory has not been proven. In any case scenario, in heavily infected patients, this microorganism could contribute to periodontal breakdown [52]. Interestingly, this species has not been commonly detected in studies using NGS techniques [21,15].

Two other species worth mentioning are *S. aureus* and *E. coli*, both classified as Moderate evidence. They followed a similar pattern to *C. sakazaki, E. faecalis*, and *S. warneri* but were less robust since some of the significance was only reached in the non-adjusted analyses.

*Aureus* secretes several toxins which are related to respiratory infections [53]. One of the pathogenic mechanisms used by this microorganism is the production of virulence factors that effectively alter specific target cell functions [54]. *S. aureus* has been observed in the oral cavity, mainly infecting root canals [55]. Souto et al. [49] found *S. aureus* elevated in patients with chronic periodontitis. Conversely, da Silva-Boghossian et al. [50] demonstrated that the frequency and counts of *S. aureus* in subgingival biofilm did not differ from sites with chronic periodontitis and periodontal health. Furthermore, other investigations have reported that *S. aureus* can be found in the subgingival biofilm regardless of the patient’s periodontal condition [56,57]. More recently, the role of *S. aureus* on polymicrobial peri-implant infections has been investigated [58,59]. Hence, additional studies evaluating *S. aureus* isolated from healthy and diseased teeth and implants are needed to clarify these controversial findings.

*Coli* is a bacterium that normally colonizes human and some animal intestines, but in some cases can cause infections, such as urinary and intestinal infections [60,61]. Like *E. faecalis*, this species has been associated with the etiology of periodontitis mostly in studies evaluating Brazilian patients [49]. Therefore, this may be another example of a specific periodontal pathogen of the Brazilian population that deserves further investigation.

The fact that *P. endondontalis* did not show any association with periodontitis in the present study was unexpected. This species has been considered as a possible periodontal pathogen in studies using nested PCR [62], Sanger sequencing [63], or pyrosequencing [19,20]. A similar situation occurred with *D. pneumosintes*, which also showed no specific association with periodontitis but had already been associated with periodontitis in previous investigations [16,21,62]. On the other hand, *F. alocis*, included in our study in the Strong evidence category, had already been suggested as a potential periodontal pathogen in several previous studies evaluating the microbiota of adults [16,19,20,63,64] and young patients with periodontitis [64; 65]. This species has also been related to xinfected root canals [66] and peri-implantitis [67].

The results of the Elimination study confirm data from previous studies suggesting that both treatments lead to improvements in periodontal clinical parameters, but when MTZ + AMX are used in combination with SRP these improvements are more robust [68–73]. The results of the 40 bacterial species from the traditional Checkerboard DNA-DNA hybridization panel underpin the clinical results and confirm the value of these species as biological markers for evaluating the effectiveness of different periodontal therapies [5,9,11,14,26] Patients treated with adjunctive MTZ+AMX showed a more beneficial biofilm composition even 1 year after antibiotic intake. This indicates that this treatment protocol is more effective than mechanical treatment alone to prevent biofilm resilience. In microbial ecology, resilience is the capacity of an ecosystem to deal with perturbation without shifting to an alternative state [74]. Most importantly, along with the benefits of these therapies, especially of the SRP+ MTZ + AMX, in the clinical parameters and the classic bacterial species, the Elimination study confirms the effect of these therapies in reducing the levels and prevalence of species that were considered as possible new periodontal pathogens. All 12 species categorized as Strong or Moderate evidence were significantly reduced by SRP+MTZ+AMX, while only 4 of them (*P. gergoviae*, *H. influenzae*, *S warneri*, and *S. ixodetis*) were significantly reduced by SRP only.

NGS analysis has increased our knowledge about bacterial communities due to a broad detection of species, including as-yet-uncultured taxa [75]. Although studies using NGS techniques are encouraged, the 40 species of the Checkerboard DNA-DNA hybridization panel continue to be consistent markers for oral dysbiosis and homeostasis. Additionally, the Checkerboard test allows the quantification of individual species, data not provided by sequencing techniques. Importantly, up until now, OMICS knowledge has not changed how we treat patients in daily practice [15]. Also, studies using meta-transcriptomic analysis to assess the metabolic functions and the virulence factors expressed by these potential novel pathogens would be fundamental to broadening our vision of the complex functionality of the oral microbiome. Thus, future standardized, large, multi-center case-control studies using multiple sequencing techniques (e.g. 16S rRNA gene sequencing, metagenomic, transcriptomics, proteomics, and metabolomics) must be encouraged.

The main limitations of this study are the low number of healthy patients evaluated and the close-ended microbial test used, which precludes the identification of uncultivated organisms. The main strength is to be the first study to combine an Association and an Elimination study and to evaluate a high number of species to weigh the evidence for their potential as new periodontal pathogens. These two experimental designs are considered the most important level of evidence when establishing a causal relationship between a microorganism and a particular infection [23].

The evaluation of the ‘new’ candidate pathogens in deep and shallow sites is also considered a strength of the study design. Previous Association studies have determined that species considered true pathogens, such as the red complex microorganisms, and newly identified pathogens are found at higher levels in deep sites of volunteers with periodontitis [3,15,76]. Even so, a continuous debate in the periodontal literature questions whether higher levels/prevalence of a microorganism in deep pockets of patients with periodontitis would configure a causal association with periodontitis. It has been speculated that this might be due to an overgrowth of these species, favored by inflammatory environmental stimuli in the periodontal pocket, such as the absence of oxygen and the large availability of nutrients for the growth of these pathogens (*e.g.*, metals, amino acids, and peptides) [77–79]. Hence, the strategy used in our study of determining the presence of microorganisms also in shallow sites (PD ≤3 mm) helps to establish a causal relationship between their presence and the onset of infection.

Nevertheless, the question of whether periodontal disease onset is triggered by the new potential pathogens identified in this study (isolated or in combination) is extremely difficult to answer in humans. This is true even for classic pathogens in prospective clinical study designs including longitudinal repeated sampling of the same patients. Those limitations strengthen the rationale for asking such questions experimentally using germ-free mouse models, for example. In such models, each species can be introduced separately or in combination and their individual and combined effects on periodontal onset and/or progression may be assessed [80].

Another point of consideration relates to the functionality of these potential new pathogens, such as the ‘pathobionts’ concept [79]. According to this theory,under conditions of disrupted homeostasis, commensal microorganisms have the potential to cause deregulated inflammation and disease . This might explain why healthy patients are colonized with some of these newly identified pathogens without developing disease [81].

Regarding the statistical analysis, it is important to highlight that concerning the shallow site data, most of the statistically significant differences were observed only by the unadjusted analysis. This was expected since the number of samples is reduced to one-third when only shallow sites are considered. Similarly, for the Elimination study, the periodontitis group (*n* = 40) was divided into two groups of 20 volunteers, which also reduces the power of the study and may make it difficult to obtain statistical significance when adjusting for multiple comparisons. Therefore, when categorizing species according to the level of evidence for periodontal pathogen status, it was important to consider the results obtained in the adjusted and unadjusted analyses for shallow site data and the Elimination study.

## Conclusions

Our findings suggest strong evidence supporting *L. rimae, C. sakazakii, P. gergoviae, E. faecalis, E. limosum, F. alocis, H. influenzae*, and *S. warneri*, and moderate evidence supporting *E. coli, F. necrophorum, S. ixodetis*, and *S. aureus* as periodontal pathogens. Together, these findings contribute to a better understanding of the etiology of periodontitis and may guide future diagnostic and interventional studies

## Supplementary Material

Supplemental Material

Supplemental Material
