# Supplementary material for: Newly identified pathogens in periodontitis: evidence from an association and an elimination study
Source: J Oral Microbiol. 2023 May 27;15(1):2213111. doi: 10.1080/20002297.2023.2213111 (PMC10228317; doi:10.1080/20002297.2023.2213111)
Supplement: Supplemental Material [file ZJOM_A_2213111_SM9313.zip › Supplementary files/Figure Supplemental 1.docx]

**Figure Supplemental 1:** Mean proportion of the 79 bacterial species evaluated in the periodontitis group. Species are listed in descending order according to proportion. In red, are the “new” species studied; in blue, are the species from the traditional Checkerboard DNA-DNA hybridization panel.
