# Supplementary figures and images for: Newly identified pathogens in periodontitis: evidence from an association and an elimination study
Source: J Oral Microbiol. 2023 May 27;15(1):2213111. doi: 10.1080/20002297.2023.2213111 (PMC10228317; doi:10.1080/20002297.2023.2213111)

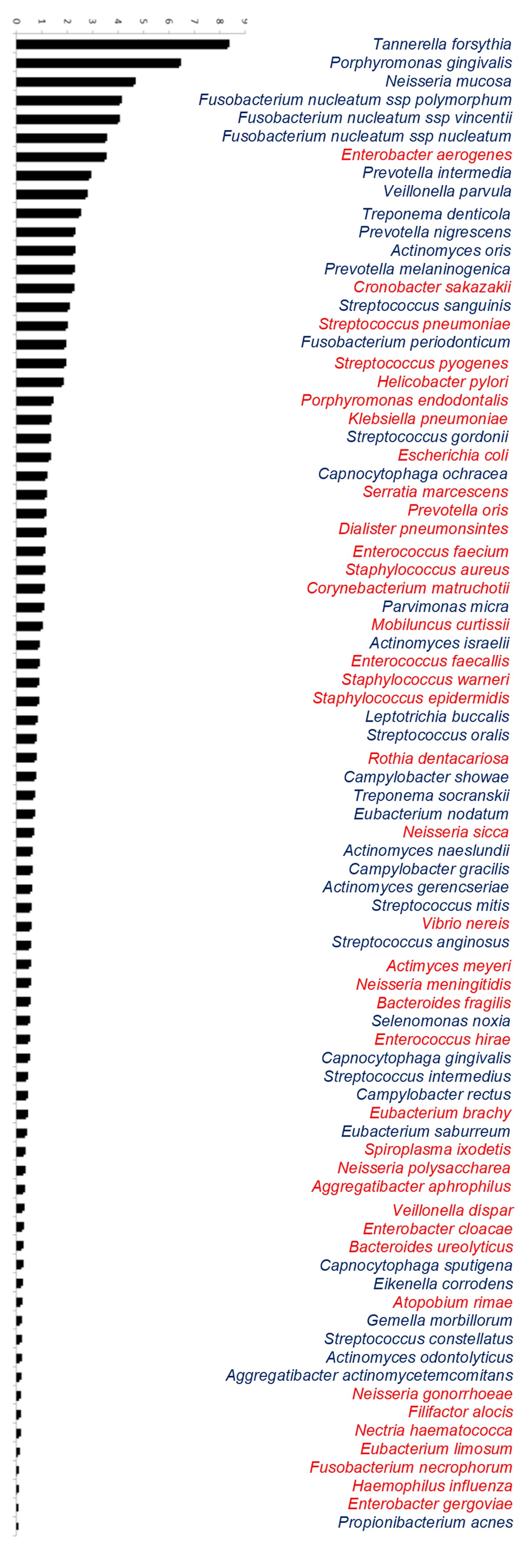

Supplement: Supplemental Material [file ZJOM_A_2213111_SM9313.zip › Supplementary files/Supplemental Figure 1.jpg]
